# The Anticonvulsant Effects of SR 57227 on Pentylenetetrazole-Induced Seizure in Mice

**DOI:** 10.1371/journal.pone.0093158

**Published:** 2014-04-01

**Authors:** Bingjin Li, Liang Wang, Zhihui Sun, Yang Zhou, Dongyuan Shao, Jing Zhao, Yunong Song, Jiayin Lv, Xue Dong, Changhong Liu, Pu Wang, Xingyi Zhang, Ranji Cui

**Affiliations:** 1 Second hospital of Jilin University, Changchun, China; 2 National Engineering Laboratory for Druggable Gene and Protein Screening Northeast Normal University, Changchun, China; 3 First hospital of Jilin University, Changchun, China; 4 China-Japan Union Hospital, Changchun, China; St. Joseph's Hospital and Medical Center, United States of America

## Abstract

Recently, studies have shown that serotonin plays an important role in the control of seizure. However, the specific role of 5-HT receptor subtypes is not yet well described, in particular that of the 5-HT_3_ receptor. The present study was aimed to investigate the role of 5-HT_3_ receptor on the pentylenetetrazole (PTZ)-induced seizure in mice. Firstly, seizure latency was significantly prolonged by a 5-HT_3_ receptor agonist SR 57227 in a dose-dependent manner. Seizure score and mortality were also decreased by SR 57227 in PTZ-treated mice. Furthermore, these anticonvulsant effects of SR 57227 were inhibited by a 5-HT_3_ receptor antagonist ondansetron. However, ondansetron alone had no effect on seizure latency, seizure score or mortality at different doses. Immunohistochemical studies have also shown that c-Fos expression was significantly increased in hippocampus (dentate gyrus, CA1, CA3 and CA4) of PTZ-treated mice. Furthermore, c-Fos expression was significantly inhibited by ondansetron in mice treated with PTZ and SR 57227. An ELISA study showed that SR 57227 attenuated the PTZ-induced inhibitory effects of GABA levels in hippocampus and cortex, and the attenuated effects of SR 57227 were antagonized by ondansetron in hippocampus but not cortex. Our findings suggest that activation of 5-HT_3_ receptor by SR 57227, which plays an important role on the control of seizure induced by PTZ, may be related to GABA activity in hippocampus. Therefore, 5-HT_3_ receptor subtype is a potential target for the treatment of epilepsy.

## Introduction

Epilepsy is one of the most common serious neurological disorders in the world. It has been reported that more than 50 million people worldwide have suffered from epilepsy, which causes substantial morbidity and mortality [1]. 5-HT receptors are widely distributed in both central and peripheral nervous system. Recently the involvement of various 5-HT receptor subtypes in seizure disorders has been described in several animal studies [2]–[4]. For example, serotonin deficiency can induce epileptic seizures while some antiepileptic drugs produce antiepileptic effects by increasing extracellular 5-HT level [2]–[4]. Furthermore, several 5-HT receptor subtypes may be relevant to epilepsy, such as 5-HT1A, 5-HT1B and 5-HT3 [2]–[5]. Particularly evidence of behavioral pharmacology showed that activation of 5-HT3 exerted the anticonvulsive effect. However, the detailed mechanism remains unknown.

Activation of 5-HT_3_ receptors significantly increased the seizure threshold induced by pentylenetetrazole (PTZ), while blockade of 5-HT_3_ receptors by granisetron proved proconvulsant [3]. Furthermore, the additive anticonvulsant effects of citalopram and morphine on PTZ-induced clonic seizures in mice were prevented by pretreatment with low and non-effective doses of a 5-HT_3_ receptor antagonist tropisetron and augmented by a 5-HT_3_ receptor agonist 1-(m-chlorophenyl)-biguanide (mCPBG) [5]. Sharma et al. [4] also reported that ondansetron showed a proconvulsive proclivity and lowered the PTZ-induced clonic seizure threshold. These findings suggest that antagonism at 5-HT_3_ receptor theoretically maybe provoke seizure development [6], and 5-HT_3_ receptor may play an important role in anticonvulsant effect. However, other studies have reported that block of 5-HT_3_ by ondansetron [7], [8] or tropisetron [9] has an anti-seizure activity on PTZ-induced seizure. Therefore, based on these differing findings as mentioned above, the main objective of the present study was to examine the role of 5-HT_3_ on the control of seizure induced by PTZ in mice.

PTZ, a γ-aminobutyric acid (GABA_A_) receptor antagonist, has been used extensively to induce seizures in animal models [10], [11]. Therefore, the main action of the PTZ-induced seizure reduces GABA levels [10]–[13]. Some evidences show that 5-HT_3_ receptor is also expressed by GABAergic neurons in hippocampus and cortex [14], [15]. Moreover, ondansetron, a 5-HT_3_ receptor antagonist, has been reported to affect GABA-activated current in experimental animals [16]. These data suggest that GABAergic neuron may be involved in the effects of 5-HT_3_ receptor on control of seizure. In addition, c-Fos expression was also induced by PTZ in the hippocampus of rodents [10], [11], [17]. C-Fos, an immediate early gene, is a marker of increase in neuronal activity. These data suggest that neuronal activity of hippocampus may be related to seizure induced by PTZ. Therefore, the present study was aimed to determine the effects of SR 57227, a 5-HT_3_ receptor agonist, on seizure latency (or score) and c-Fos expression and GABA levels in relevant regions of the brain of mice with PTZ-induced seizure.

## Materials and Methods

### Animals

Male ICR mice weighing 23 ± 2 g (Jilin University, Jilin, China) were used in the current study. Animals were housed in the standard conditions, including controlled temperature (22 ± 1 °C), 12 h dark/12 h light cycle. These mice were divided in polycarbonate cages with access to food and water. Each treatment group consisted of 8–10 animals and each mouse was used only once. All experiments were conducted between 9:00 am and 16:00 pm.

### Ethics Statement

The study was conducted in accordance with the Guide for the Care and Use of Laboratory Animals published by National Institutes of Health and with the recommendations and approval of the Ethics Committee on Animal Experiments of the Northeast Normal University. All efforts were made to minimize suffering.

### Drugs

The following drugs were used: pentylenetetrazole (Sigma, St. Louis, USA), SR 57227 hydrochloride and ondansetron hydrochloride (Tocris, Ellisvlle, MO), pentobarbital sodium and sodium valproate (Sigma, St. Louis, MO). All the drugs were dissolved in saline. Dose of the SR 57227 (1, 5, 10 mg/kg, i.p.) and ondansetron (0.2 mg/kg, i.p.) were chosen based on previous reports from our group and others [3], [8], [10], [18]. Ondansetron and SR 57227 were injected intraperitoneally (i.p.) 45 minutes and 30 minutes before administration of PTZ, respectively.

### Seizure latency, score and mortality

All mice were randomized into five groups (n  =  8), and injected PTZ (60, 65, 70, 80 and 90 mg/kg, i.p.) to determine the optimal dosage for the PTZ-induced acute seizure model (data not shown here). Our preliminary data have shown that PTZ at a single dose of 65 mg/kg produced seizure, a phenotype reflected by an increase in the seizure score, showing more than three consecutive tonic-clonic seizures. However, higher doses (70, 80 and 90 mg/kg) caused the higher mortality rate. We therefore applied 90 mg/kg PTZ to induce mortality in mice. The animals were observed for 30 min after the injection and latency to the first generalized tonic-clonic seizures was measured [11]. Seizure assessments were carried out by an experimenter blind to animal information.

Behavioral changes were monitored for 30 min after PTZ (65 mg/kg, i.p.) injection. The seizure behavior was classified as follows [19]: stage 0, no response; stage 1, eat and facial twitching; stage 2, myoclonic body jerks; stage 3, forelimb clonus, rearing; stage 4, clonic convulsions, turn onto the side; and stage 5, generalized clonic convulsions, turn onto the back. The latencies to the onset of myoclonic jerks and clonic seizures were evaluated for 30 min after the PTZ injection [8]. In addition, in mortality testing all mice were randomized into 8 groups (n  =  8–10). Mortality was also recorded to minimize suffering after the injection of high-dose PTZ (90 mg/kg, i.p.). The animals were humanely euthanized by CO_2_ inhalation at end of study when animal vital signs were lost. Every effort was made to minimize suffering, such as reducing the number of animals and test time, avoiding painful stimulation and euthanasia.

### C-Fos immunohistochemistry

All mice were randomized into six groups (n  =  6–7). Animals were killed 2 h after injection of PTZ, the brains were removed rapidly, flash frozen in isopentane and stored at −80°C. Immunohistochemistry for c-Fos protein was carried out as described by previous reports from our group and others [17], [20]. Serial coronal sections from forebrain to brainstem, 30 μm thick, were cut in a cryostat. Free-floating sections were rinsed in 0.05 M phosphate buffered saline (PBS; pH 7.4) and then incubated with 0.6% hydrogen peroxide in PBS to remove endogenous peroxidase activity. After rinsing again in PBS, the sections were incubated with primary antibody (1:1000 dilutions in PBS containing 0.3% Triton X-100, 0.05% sodium azide, and 2% normal goat serum) for 72 h at 4°C. The c-Fos antibody (SC-52; Santa Cruz Biotechnology, Santa Cruz, CA) was a rabbit polyclonal antibody raised against a peptide mapping at the amino terminus of human c-Fos p62. The sections were than rinsed and incubated with a secondary antibody [biotinylated goat anti-rabbit IgG (Vector Laboratories, Burlingame, CA) 1:400 dilution in PBS with 0.3% Triton X-100] for 75 min at room temperature. After being rinsed, the sections were transferred into PBS containing 0.4% avidin-biotinylated horseradish peroxidase complex (Vector Laboratories, Burlingame, CA) and incubated for another 75 min. Following successive washes in PBS and 0.2 M sodium acetate buffer (pH 6.0), the reaction product was then visualized using a glucose oxidase-diaminobenzidine-nickel method described by our report [17], [20]. The reaction was terminated through washing the sections in sodium acetate buffer. Thereafter, the sections were mounted onto chrome-alum-gelatin-coated slides from 0.05 M PBS. After being air-dried, the sections were counterstained with neutral red, dehydrated through a graded alcohol series, cleared in xylene and finally cover slipped.

The c-Fos-positive neurons were identified by the presence of dense immunohistochemical staining within the dentate gyrus of the hippocampus and medial prefrontal cortex under a light microscope. The counting was performed bilaterally on a minimum of two representative sections per level. The positive cells were counted under a magnification of × 200 from three brain regions.

### GABA ELISA

All mice were randomized into six groups (n  =  7-10). Brain GABA levels were measured using a mouse GABA ELISA kit (Nanjing Jiancheng Bioengineering Institute, Nanjing, China) according to the manufacturer's instructions. Briefly, at the end of the treatment, animals were sacrificed by decapitation. The brains were quickly removed and dissected on ice into the hippocampus and cortex. Samples were frozen at − 80 °C before homogenization, and a centrifuged supernatant of each sample was used to measure the GABA level by using the GABA ELISA kit. For more detailed information on the ELISA method refer to our report and previous reports [21], [22].

### Statistical analysis

The data were analyzed using one-way analysis of variance (one-way ANOVA). When significant differences were obtained, post-hoc comparisons within logical sets of means were performed using Tukey's test. The mortality rate was analyzed using Fisher's exact test. *P* values less than 0.05 were considered statistically significant.

## Results

### Effects of SR 57227 and ondansetron on seizure latency and seizure score in PTZ-treated mice

**Figure 1:**
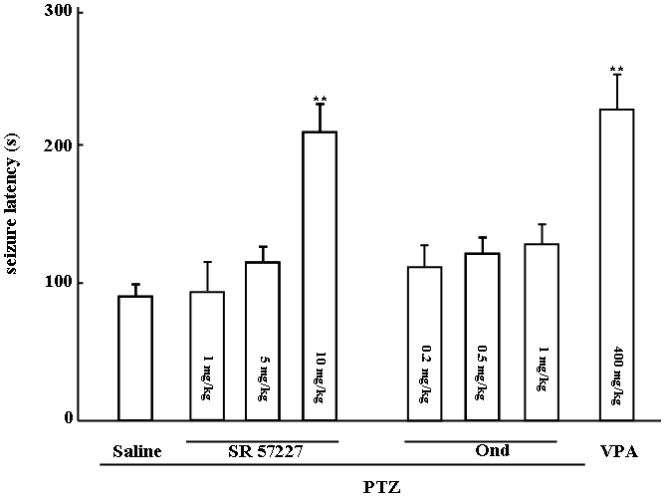
Effects of SR 57227 and ondansetron on seizure latency in PTZ-treated mice. PTZ: pentylenetetrazole (65 mg/kg, i.p.); SR 57227: 1, 5 and 10 mg/kg, i.p.; Ond: ondansetron (0.2, 0.5 and 1 mg/kg, i.p.); VPA: sodium valproate (400 mg/kg, p.o.). Columns represent the mean ± S.E.M. n  =  8–10. ** *P*<0.01 vs saline group.

**Figure 2:**
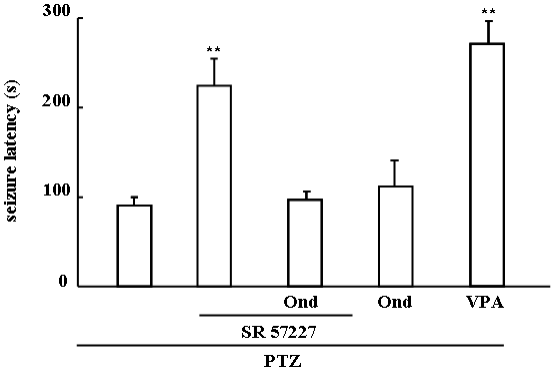
Effects of ondansetron on SR 57227-induced prolonged seizure latency. PTZ: pentylenetetrazole (65 mg/kg, i.p.); SR 57227: 10 mg/kg, i.p; Ond: ondansetron (0.2 mg/kg, i.p.); VPA: sodium valproate (400 mg/kg, p.o.). Columns represent the mean ± S.E.M. n  =  8–10. ** *P*<0.01 vs PTZ group.

Effects of SR 57227 and ondansetron on seizure latency in PTZ-induced seizure mice are shown in Fig.1. The seizure latency was significantly prolonged by high-dose SR 57227 (10 mg/kg, i.p. F (4, 39)  =  25.159, *P*<0.01) but not affected by ondansetron (0.2, 0.5 and 1.0 mg/kg, i.p.). However, the effects of SR 57227 on seizure latency were significantly inhibited by ondansetron in Fig.2. Furthermore, VPA (400 mg/kg, p.o.), a standard anticonvulsant drug, also significantly increased the seizure latency compared to control group (*P*<0.01).

In table 1 the effects of SR 57227 and ondansetron on seizure score in PTZ-treated mice were shown. Compared to PTZ-treated group, seizure score was markedly inhibited by high-dose SR 57227 (10 mg/kg, F (3, 30)  =  14.034, *P*<0.05). Furthermore, the effects of SR 57227 (10 mg/kg, i.p.) on seizure score was attenuated by ondansetron (0.2 mg/kg, i.p.) in PTZ-treated mice. However, ondansetron alone have no effect on seizure score in PTZ-treated mice [F (3, 31)  =  0.003, *P*>0.05]. In addition, seizure score was also significantly inhibited by VPA in PTZ-treated mice.

**Table 1:** Effects of SR 57227 and ondansetron on PTZ-induced seizure score in mice.

Treatment	Seizure score
PTZ	4.85±0.14
PTZ + SR (1 mg/kg)	4.23±0.13
PTZ + SR (5 mg/kg)	3.82±0.43
PTZ + SR (10 mg/kg)	2.09±0.22*
PTZ + Ond (0.2 mg/kg)	4.86±0.10
PTZ + Ond (0.5 mg/kg)	4.77±0.15
PTZ + Ond (1 mg/kg)	4.81±0.26
PTZ + SR (10 mg/kg) + Ond (0.2 mg/kg)	4.75±0.32
PTZ + VPA (400 mg/kg)	1.47±0.21*

SR: SR 57227 (1, 5, and 10 mg/kg, i.p); PTZ: pentylenetetrazole (65 mg/kg, i.p); Ond: ondansetron (0.2, 0.5 and 1 mg/kg, i.p.). VPA: sodium valproate (400 mg/kg, p.o.). n  =  8–10 per group. * *P*<0.05, vs PTZ group.

### Effects of SR 57227 and ondansetron on mortality in PTZ-treated mice

In the mortality studies, we observed 100% mortality in mice with PTZ (90 mg/kg, i.p.) alone within 30 min. Animals died as a direct result of the intervention. Mortality was monitored by video camera only for 6 h to minimize suffering in other groups. Mice with video monitoring were checked once every 30 min. In table 2 the mortality induced by high-dose PTZ (90 mg/kg, i.p.) were significantly inhibited by high-dose SR 57227 (10 mg/kg, i.p) and VPA (400 mg/kg, p.o.) but not ondansetron (0.2, 0.5 and 1.0 mg/kg, i.p.). Furthermore, the inhibitory effects of SR 57227 on mortality induced by PTZ were also blocked by ondansetron (0.2 mg/kg, i.p.). However, ondansetron did not alter mortality induced by high-dose PTZ in mice.

**Table 2:** Effects of SR 57227 and ondansetron on PTZ-induced mortality in mice.

Treatment	Mortality	Mortality%
PTZ	10/10	100
PTZ + SR (1 mg/kg)	8/8	100
PTZ + SR (5 mg/kg)	6/8	75
PTZ + SR (10 mg/kg)	3/10**	30
PTZ + Ond (0.2 mg/kg)	9/10	90
PTZ + Ond (0.5 mg/kg)	8/9	88.9
PTZ + Ond (1 mg/kg)	8/10	80
PTZ + SR (10 mg/kg) + Ond (0.2 mg/kg)	8/9	88.9
PTZ + VPA (400 mg/kg)	2/9**	22.2

SR: SR 57227 (1, 5, and 10 mg/kg, i.p.); PTZ: pentylenetetrazole (90 mg/kg, i.p.); Ond: ondansetron (0.2, 0.5 and 1 mg/kg, i.p.). VPA: sodium valproate (400 mg/kg, p.o.). n  =  8–10 per group. ** *P*<0.01 (Fisher's exact test), vs PTZ group.

### Effects of SR 57227 and ondansetron on c-Fos expression in hippocampus of PTZ-treated mice

**Figure 3:**
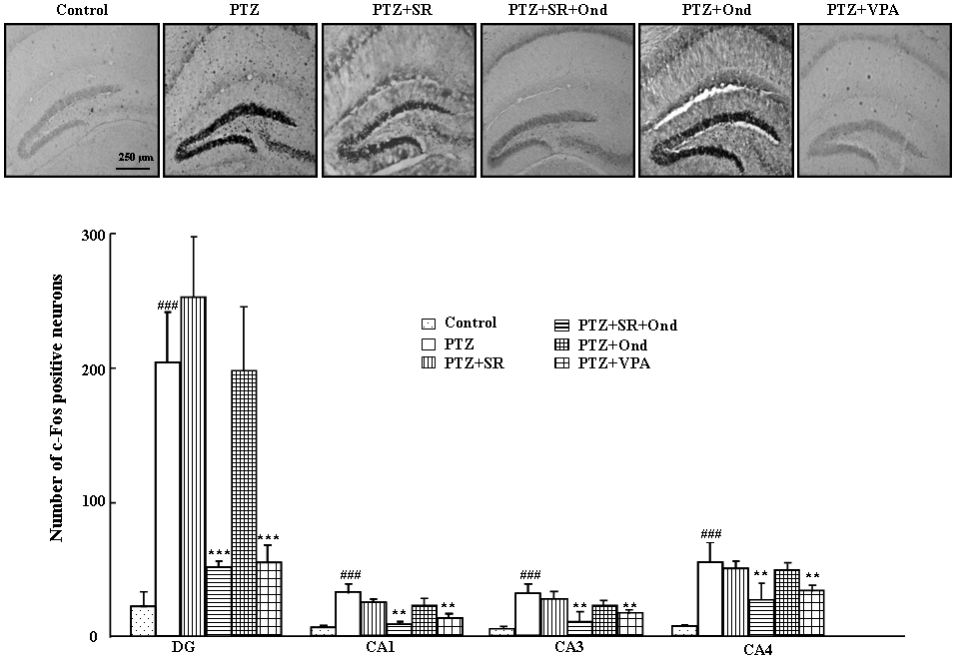
Effects of ondansetron and SR 57227 on c-Fos expression in hippocampus. PTZ: pentylenetetrazole (65 mg/kg, i.p.); SR 57227: 10 mg/kg, i.p; Ond: ondansetron (0.2 mg/kg, i.p.); VPA: sodium valproate (400 mg/kg, p.o.). DG: dentate gyrus; Columns represent the mean ± S.E.M. n  =  6–7. ### *P*<0.001 vs control group; ** *P*<0.01, *** *P*<0.001 vs PTZ group.

Fig.3 shows that the effects of ondansetron and SR 57227 on c-Fos expression in hippocampus of PTZ-treated mice. C-Fos expression was examined in the dentate gyrus, CA1, CA3 and CA4 of the hippocampus. PTZ (65 mg/kg, i.p.) significantly increased the c-Fos expression (*P*<0.001), and VPA (400 mg/kg, p.o.) but not SR 57227 (10 mg/kg, i.p.) significantly decreased PTZ-induced c-Fos expression in these regions of hippocampus. However, the c-Fos expression was significantly inhibited by ondansetron in mice treated with SR 57227 and PTZ compared to the PTZ-treated group alone (*P*<0.001).

### Effects of SR 57227 and ondansetron on GABA levels in hippocampus and cortex of PTZ-treated mice

**Figure 4:**
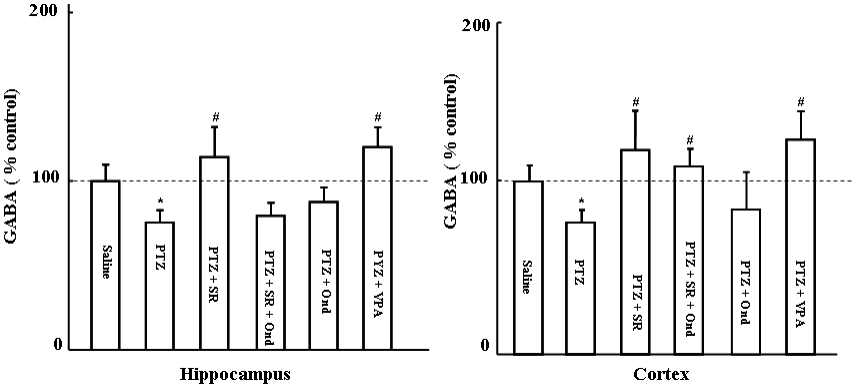
Effects of SR 57227 on GABA levels, normalized to control group, in hippocampus and cortex. PTZ: pentylenetetrazole (65 mg/kg, i.p.); SR 57227: 10 mg/kg, i.p; Ond: ondansetron (0.2 mg/kg, i.p.); VPA: sodium valproate (400 mg/kg, p.o.). Values are expressed as mean + SEM. n  =  7–10. * *P*<0.05 vs saline group; # *P*<0.05 vs PTZ group.

Fig. 4 shows the effects of SR 57227 and ondansetron on GABA levels in hippocampus and cortex of PTZ-treated mice. GABA levels were significantly inhibited by PTZ (65 mg/kg, i.p.) in both hippocampus and cortex (hippocampus, *P*<0.05; cortex, *P*<0.05). Furthermore, reduction of GABA levels was attenuated by SR 57227 in PTZ-treated mice (*P*<0.01). However, the effects of SR 57227 on GABA levels in hippocampus but not cortex were blocked by ondansetron (0.2 mg/kg, i.p.).

## Discussion

In the present study seizure latency was significantly prolonged by acute SR 57227 in a dose-dependent manner, and the seizure score and mortality were also decreased by SR 57227. Furthermore, these anticonvulsant effects were blocked by ondansetron. These findings suggest that activation of 5-HT_3_ receptor may play an important role on control of seizure induced by PTZ. However, previous studies have also shown that acute administration of SR 57227 (0.3–3 mg/kg, i.p.) did not alter seizure threshold [23] or severity [24]. Gholipour et al. [3] also reported that SR 57227 (10 mg/kg, i.p.) increased the seizure threshold in PTZ-treated mice. These results also reveal that activation of 5-HT_3_ induced by high-dose but not low-dose of SR 57227 may be related to anticonvulsant effect in PTZ-treated mice. Interestingly, another 5-HT_3_ receptor antagonist granisetron (10 mg/kg, i.p) proved proconvulsant [3]. However, in our study, ondansetron (0.2, 0.5 and 1 mg/kg, i.p) alone had no anticonvulsant effect in PTZ-treated mice. Consistent with these findings, additional evidence also shows that even a broader dose range of acute ondansetron (0.5–2 mg/kg, i.p) had no effect on seizure induced by PTZ but inhibited electroshock-induced seizure threshold [8]. Based on these different 5-HT_3_ antagonists or dosages of 5-HT_3_ antagonist, it was difficult to make direct comparisons among these studies. However, these data suggest at a minimum that the effect of 5-HT_3_ receptor antagonist on seizure was more complex compared to that of 5-HT_3_ agonist on seizure induced by PTZ, and different effects of various antagonists are likely to depend not only on dosage but also on the type of seizure. In addition, the 5-HT_3_ receptor agonists (SR57227 and mCPBG) exert the anticonvulsive effect while 5-HT_3_ receptor antagonists (granisetron and tropistron) lower the threshold of PTZ-induced clonic seizure [3], [9]. Combined with our findings, these data suggest that activation of 5-HT_3_ receptors may be involved in seizure induced by PTZ, and 5-HT_3_ receptor subtype is a potential target for the treatment of epilepsy.

Previous studies also showed that PTZ significantly increased c-Fos expression in many brain regions [25], [26]. However, Hippocampus is most widely analyzed as a representative brain region in PTZ-induced seizure mice [11], [17], [25]–[27]. In our immunohistochemical study c-Fos expression was also significantly increased by PTZ in the dentate gyrus, CA1, CA3 and CA4 of the hippocampus. Furthermore, c-Fos expression was inhibited by ondansetron in mice treated with PTZ and SR 57227. Consistent with behavioral data, these results indicate that neuronal activity in hippocampus may be involved in the anticonvulsant effect of SR 57227 on control of seizure induced by PTZ. In addition, ondansetron alone had no effect on c-Fos expression in hippocampus of PTZ-treated mice. This finding is also supported by our behavioral data. These results may indicate that low dosage of ondansetron (0.2 mg/kg, i.p) alone is too low to affect c-Fos expression in hippocampus of PTZ-treated mice. Another possibility is that ondansetron may be more sensitive to activation of 5-HT_3_ receptor induced by SR 57227 in PTZ-treated mice compared to PTZ-treated group without SR 57227.

In addition, the main action of the PTZ-induced seizure is reducing GABA levels [12], [13]. The effects of SR 57227 on GABA levels in cortex and hippocampus were also evaluated in PTZ-induced seizure mice. In the present study GABA levels were significantly inhibited by PTZ, and these inhibitions were also attenuated by SR 57227 in both hippocampus and cortex. However, ondansetron reversed the effect of SR 57227 on GABA levels in hippocampus but not cortex of PTZ-treated mice. These data reveal that GABA in hippocampus is more responsible for the anticonvulsant effects of SR 57227 on control of seizure induced by PTZ compared to that in cortex. Our data is also supported by a previous study that 5-HT_3_ receptor could modulate a GABAergic-mediated seizure threshold [16], [28] or GABAergic neuron in hippocampus [14], [15]. However, in this study ondansetron alone did not affect GABA levels in both hippocampus and cortex of PTZ-induced seizure mice. Here we applied ondansetron in lower dose (0.2 mg/kg, i.p). Therefore, it may be unable to show any effect on c-Fos in PTZ-treated mice.

VPA, as a standard anticonvulsant drug, has anticonvulsant effects on seizure induced by PTZ. Previous studies showed that VPA is able to increase brain GABA levels via various mechanisms, including blocking GABA reuptake, inhibiting the enzymes that break down GABA, and enhanced GABA release from nerve terminals [29]. In our study the inhibitory effects of GABA levels were also reversed by VPA in hippocampus and cortex of PTZ-treated mice.

In conclusion, our findings suggest that activation of 5- HT_3_ receptor, plays an important role in the control of seizure induced by PTZ, may be related to GABAergic neuronal activity in hippocampus, and that the 5-HT_3_ receptor subtype is a potential target for the treatment of epilepsy.

## References

[1] Hirose S, Okada M, Kaneko S, Mitsudome A (2000) Are some idiopathic epilepsies disorders of ion channels? A working hypothesis. Epilepsy Res 41: 191–204. 10962210 10.1016/s0920-1211(00)00141-8

[2] Bagdy G, Kecskemeti V, Riba P, Jakus R (2007) Serotonin and epilepsy. J Neurochem 100: 857–873. 17212700 10.1111/j.1471-4159.2006.04277.x

[3] Gholipour T, Ghasemi M, Riazi K, Ghaffarpour M. Dehpour AR (2010) Seizure susceptibility alteration through 5-HT(3) receptor: modulation by nitric oxide. Seizure 19: 17–22. 19942458 10.1016/j.seizure.2009.10.006

[4] Sharma A, Raina V (2001) Generalised seizures following ondansetron. Ann Oncol 12: 131–132. 10.1023/a:1008394422101 11249042

[5] Bahremand A, Payandemehr B, Rahimian R, Ziai P, Pourmand N, et al (2011) The role of 5-HT(3) receptors in the additive anticonvulsant effects of citalopram and morphine on pentylenetetrazole-induced clonic seizures in mice. Epilepsy Behav 21: 122–127. 21531632 10.1016/j.yebeh.2011.03.010

[6] Fakhfouri G, Rahimian R, Ghia JE, Khan WI, Dehpour AR (2012) Impact of 5-HT_3_ receptor antagonists on peripheral and central diseases. Drug Discov Today 17: 741–747. 22390946 10.1016/j.drudis.2012.02.009

[7] Puthuran GJ, Patil PA, Majagi SI (2009) Effect of ondansetron on convulsions and its interactions with phenytoin. Pharmacologyonline 3: 463–469.

[8] Jain S, Agarwal NB, Mediratta PK, Sharma KK (2012) Evaluation of anticonvulsant and nootropic effect of ondansetron in mice. Hum Exp Toxicol 31: 905–912. 22354082 10.1177/0960327112436406

[9] Payandemehr B, Bahremand A, Rahimian R, Ziai P, Amouzegar A, et al (2012) 5-HT(3) receptor mediates the dose-dependent effects of citalopram on pentylenetetrazole-induced clonic seizure in mice: involvement of nitric oxide. Epilepsy Res 101: 217–227. 22578701 10.1016/j.eplepsyres.2012.04.004

[10] Erdtmann-Vourliotis M, Riechert U, Mayer P, Grecksch G, Höllt V (1998) Pentylenetetrazole (PTZ)-induced c-Fos expression in the hippocampus of kindled rats is suppressed by concomitant treatment with naloxone. Brain Res 792: 299–308. 9593956 10.1016/s0006-8993(98)00159-0

[11] Chen CR, Tan R, Qu WM, Wu Z, Wang Y, et al (2011) Magnolol, a major bioactive constituent of the bark of Magnolia officinalis, exerts antiepileptic effects via the GABA/benzodiazepine receptor complex in mice. Br J Pharmacol 164: 1534–1546. 21518336 10.1111/j.1476-5381.2011.01456.x PMC3221106

[12] Psarropoulou C, Matsokis N, Angelatou F, Kostopoulos G (1994) Pentylenetetrazol-induced seizures decrease gamma-aminobutyric acid-mediated recurrent inhibition and enhance adenosine-mediated depression. Epilepsia 35: 12–19. 8112233 10.1111/j.1528-1157.1994.tb02906.x

[13] Walsh LA, Li M, Zhao TJ, Chiu TH, Rosenberg HC (1999) Acute pentylenetetrazol injection reduces rat GABAA receptor mRNA levels and GABA stimulation of benzodiazepine binding with No effect on benzodiazepine binding site density. J Pharmacol Exp Ther 289: 1626–33. 10336561

[14] Morales M, Bloom FE (1997) The 5-HT3 receptor is present in different subpopulations of GABAergic neurons in the rat telencephalon. J Neurosci 17: 3157–3167. 9096150 10.1523/JNEUROSCI.17-09-03157.1997 PMC6573651

[15] Puig MV, Santana N, Celada P, Mengod G, Artigas F (2004) In vivo excitation of GABA interneurons in the medial prefrontal cortex through 5-HT3 receptors. Cereb Cortex 14: 1365–75. 15166106 10.1093/cercor/bhh097

[16] Ye JH, Hunt T, Wu WH, McArdle JJ (1997) Ondansetron modulates GABA(A) current of rat central nervous system neurons. Eur J Pharmacol 337: 87–94. 9389385 10.1016/s0014-2999(97)01279-x

[17] Li B, Tang F, Wang L, Liu L, Zhao J, et al (2013) Anticonvulsant effects of fuzi total alkaloid on pentylenetetrazole-induced seizure in mice. J Pharmacol Sci 123: 195–8. 24096829 10.1254/jphs.13057sc

[18] Cui R, Suemaru K, Li B, Kohnomi S, Araki H (2009) Tropisetron attenuates naloxone-induced place aversion in single-dose morphine-treated rats: role of alpha7 nicotinic receptors. Eur J Pharmacol 609: 74–7. 19374878 10.1016/j.ejphar.2008.12.051

[19] Watanabe Y, Kaida Y, Fukuhara S, Takechi K, Uehara T, et al (2011) Participation of metabotropic glutamate receptors in pentetrazol-induced kindled seizure. Epilepsia 52: 140–50. 21054350 10.1111/j.1528-1167.2010.02764.x

[20] Li B, Suemaru K, Kitamura Y, Gomita Y, Araki H, et al (2013) Imipramine-induced c-Fos expression in the medial prefrontal cortex is decreased in the ACTH-treated Rats. J Biochem Mol Toxicol 2013 27: 486–491. 10.1002/jbt.21510 23922220

[21] Li B, Zhao J, Lv J, Tang F, Liu L, et al (2013) Additive antidepressant-like effects of fasting with imipramine via modulation of 5-HT2 receptors in the mice. Prog Neuropsychopharmacol Biol Psychiatry 48C: 199–206. 10.1016/j.pnpbp.2013.08.015 24036107

[22] Miraucourt LS, Silva JS, Burgos K, Li J, Abe H, et al (2012) GABA expression and regulation by sensory experience in the developing visual system. PLoS One 7: e29086. 22242157 10.1371/journal.pone.0029086 PMC3252287

[23] Bachy A, Heaulme M, Giudice A, Michaud JC, Lefevre IA, et al (1993) SR 57227A: a potent and selective agonist at central and peripheral 5-HT3 receptors in vitro and in vivo. Eur J Pharmacol 237: 299–309. 7689975 10.1016/0014-2999(93)90282-m

[24] Watanabe K, Minabe Y, Ashby Jr CR, Katsumori H (1998) Effect of acute administration of various 5-HT receptor agonists on focal hippocampal seizures in freely moving rats. Eur J Pharmacol 350: 181–188. 9696406 10.1016/s0014-2999(98)00255-6

[25] André V, Pineau N, Motte JE, Marescaux C, Nehlig A (1998) Mapping of neuronal networks underlying generalized seizures induced by increasing doses of pentylenetetrazol in the immature and adult rat: a c-Fos immunohistochemical study. Eur J Neurosci 10: 2094–2106. 9753096 10.1046/j.1460-9568.1998.00223.x

[26] Szyndler J, Maciejak P, Turzyńska D, Sobolewska A, Taracha E, et al (2009) Mapping of c-Fos expression in the rat brain during the evolution of pentylenetetrazol-kindled seizures. Epilepsy Behav 16: 216–224. 19713157 10.1016/j.yebeh.2009.07.030

[27] Li M, Kang R, Shi J, Liu G, Zhang J (2013) Anticonvulsant activity of B2, an adenosine analog, on chemical convulsant-induced seizures. PLoS One 8: e67060. 23825618 10.1371/journal.pone.0067060 PMC3692431

[28] Nichols RA, Mollard P (1996) Direct observation of serotonin 5-HT3 receptor-induced increases in calcium levels in individual brain nerve terminals. J Neurochem 67: 581–592. 8764583 10.1046/j.1471-4159.1996.67020581.x

[29] Davis LL, Ryan W, Adinoff B, Petty F (2000) Comprehensive review of the psychiatric uses of valproate. J Clin Psychopharmacol 20: 1S–17S. 10646685 10.1097/00004714-200002001-00001

